# Error and Timeliness Analysis for Using Machine Learning to Predict Asthma Hospital Visits: Retrospective Cohort Study

**DOI:** 10.2196/38220

**Published:** 2022-06-08

**Authors:** Xiaoyi Zhang, Gang Luo

**Affiliations:** 1 Department of Biomedical Informatics and Medical Education University of Washington Seattle, WA United States

**Keywords:** asthma, machine learning, clinical decision support, forecasting, patient care management, healthcare outcome, emergency department, health outcome, prediction model

## Abstract

**Background:**

Asthma hospital visits, including emergency department visits and inpatient stays, are a significant burden on health care. To leverage preventive care more effectively in managing asthma, we previously employed machine learning and data from the University of Washington Medicine (UWM) to build the world’s most accurate model to forecast which asthma patients will have asthma hospital visits during the following 12 months.

**Objective:**

Currently, two questions remain regarding our model’s performance. First, for a patient who will have asthma hospital visits in the future, how far in advance can our model make an initial identification of risk? Second, if our model erroneously predicts a patient to have asthma hospital visits at the UWM during the following 12 months, how likely will the patient have ≥1 asthma hospital visit somewhere else or ≥1 surrogate indicator of a poor outcome? This work aims to answer these two questions.

**Methods:**

Our patient cohort included every adult asthma patient who received care at the UWM between 2011 and 2018. Using the UWM data, our model made predictions on the asthma patients in 2018. For every such patient with ≥1 asthma hospital visit at the UWM in 2019, we computed the number of days in advance that our model gave an initial warning. For every such patient erroneously predicted to have ≥1 asthma hospital visit at the UWM in 2019, we used PreManage and the UWM data to check whether the patient had ≥1 asthma hospital visit outside of the UWM in 2019 or any surrogate indicators of poor outcomes. Such surrogate indicators included a prescription for systemic corticosteroids during the following 12 months, any type of visit for asthma exacerbation during the following 12 months, and asthma hospital visits between 13 and 24 months later.

**Results:**

Among the 218 asthma patients in 2018 with asthma hospital visits at the UWM in 2019, 61.9% (135/218) were given initial warnings of such visits ≥3 months ahead by our model and 84.4% (184/218) were given initial warnings ≥1 day ahead. Among the 1310 asthma patients in 2018 who were erroneously predicted to have asthma hospital visits at the UWM in 2019, 29.01% (380/1310) had asthma hospital visits outside of the UWM in 2019 or surrogate indicators of poor outcomes.

**Conclusions:**

Our model gave timely risk warnings for most asthma patients with poor outcomes. We found that 29.01% (380/1310) of asthma patients for whom our model gave false-positive predictions had asthma hospital visits somewhere else during the following 12 months or surrogate indicators of poor outcomes, and thus were reasonable candidates for preventive interventions. There is still significant room for improving our model to give more accurate and more timely risk warnings.

**International Registered Report Identifier (IRRID):**

RR2-10.2196/5039

## Introduction

### Background

Over 262 million people in the world have asthma [1]. In the United States, around 7.8% of people have asthma, which leads to 1.6 million emergency department (ED) visits, 179,000 inpatient stays [2], and an aggregate medical cost of US $50.3 billion annually [3]. A main goal in asthma management is to curtail asthma hospital visits, ie, ED visits and inpatient stays for asthma. Part of the state of the art for achieving this goal is to implement a predictive model to find patients who are at significant risk of having asthma hospital visits in the future. If deemed high risk, a patient can be considered for enrollment in a care management program to receive preventive interventions. Then a care manager regularly follows up with the patient to monitor the patient’s asthma control status, alter the patient’s asthma medications as the need arises, and help book relevant services. This approach is employed by many health care systems, such as Intermountain Healthcare, the University of Washington Medicine (UWM), and Kaiser Permanente Northern California [4], along with many health plans, such as the health plans in 9 of 12 urban communities [5]. When used properly, this approach can curtail asthma hospital visits by up to 40% [5-9].

A care management program typically accommodates no more than 3% of patients due to capacity constraints [10]. To optimize the efficacy of such programs, we recently employed extreme gradient boosting (XGBoost) [11], a machine learning algorithm, and the UWM data to build the world’s most accurate model to forecast which asthma patients will have asthma hospital visits during the following 12 months [12]. Our model obtained an area under the receiver operating characteristic curve of 0.902, a specificity of 90.91% (13,115/14,426 patients), a sensitivity of 70.2% (153/218 patients), a positive predictive value of 10.45% (153/1464 patients), a negative predictive value of 99.51% (13,115/13,180 patients), and an accuracy of 90.6% (13,268/14,644 patients) [12]. Compared with every prior model for this prediction task [4,13-26], our model improved the area under the receiver operating characteristic curve by ≥10%.

### Objectives

Currently, two questions remain regarding our model’s performance. First, for a patient who will have asthma hospital visits in the future, how far in advance can our model make an initial identification of risk? Since any preventive intervention requires sufficient time to take effect [27,28], a model should identify the risk as early as possible to provide preventive interventions in time to avoid a poor outcome. Second, if our model erroneously predicts a patient to have ≥1 asthma hospital visit at the UWM during the following 12 months, how likely will the patient have ≥1 asthma hospital visit at a facility outside of the UWM or ≥1 surrogate indicator of a poor outcome? As our model was trained on the UWM data, it can only predict future asthma hospital visits at the UWM. The goal of this work was to answer these two questions. Part of the analysis that we conducted to answer the second question has previously been published as an abstract at the 2022 American Academy of Allergy, Asthma & Immunology Annual Meeting [29].

## Methods

### Study Elements Reused From Previous Work

The following parts were reused from our prior paper on model building using the UWM data [12]: patient cohort, features, prediction target, cutoff point for conducting binary classification, training set, test set, and predictive model.

### Ethics Approval

The institutional review board of the UWM approved this retrospective cohort study (STUDY00000118).

### Patient Cohort

As the biggest academic health care system in Washington State, the UWM maintains an enterprise data warehouse that stores clinical and administrative data from 12 clinics and 3 hospitals for adults. The patient cohort was composed of every adult asthma patient ≥18 years old who received care at any of the 15 UWM facilities between 2011 and 2018. A patient was deemed to have asthma in a given year if the patient’s visit billing data in that year included ≥1 asthma diagnosis code according to the International Classification of Diseases (ICD) tenth revision (ie, code J45.x) or ninth revision (ie, code 493.1x, 493.0x, 493.9x, or 493.8x) [13,30]. This asthma case-finding method has been shown to strike the best balance between sensitivity and positive predictive value among several rule-based asthma case-finding methods, does not require the patient to have >1 year of historical data, and is suited for use in population health management [30]. Patients who died during that year were excluded.

### Data Sets

Two data sets were used. The first data set was retrieved from the UWM’s enterprise data warehouse. This data set held structured administrative and clinical data for visits by the patient cohort to the 15 UWM facilities from 2011 to 2020. The second data set came from a commercial product, PreManage (Collective Medical Technologies Inc) [31]. This data set contained structured visit and diagnosis data for ED visits and inpatient stays during 2019 by our patient cohort at every hospital in Washington State, as well as at many other American hospitals outside of Washington State.

### Overview of Our Predictive Model

#### Prediction Target, Training Set, and Test Set

For an asthma patient at a given time point, the prediction target was whether the patient would have ≥1 asthma hospital visit during the following 12 months. The prediction was made based on the patient’s data up to that time point. An asthma hospital visit was defined as an ED visit or an inpatient stay with a principal diagnosis of asthma (ICD tenth revision code J45.x or ICD ninth revision code 493.1x, 493.0x, 493.9x, or 493.8x). During model training and testing, for each patient with asthma in a given year, we used the data of that patient by the end of the year to predict the outcome of the patient in the following 12 months [12]. Since the prediction target was in the following 12 months, the UWM data between 2011 and 2019 provided 8 years of effective data for model training and testing. The effective data from 2011 to 2017 were used as the training set for training our predictive model, and the effective data from 2018 were used as the test set for testing our model. To answer our study’s two questions, we focused on the asthma patients in the test set (ie, the asthma patients in 2018), and examined the predictions made by our model for these patients. For the asthma patients in 2018 who were erroneously predicted to have asthma hospital visits at the UWM in 2019, the UWM data from 2020 were used to compute one of the surrogate indicators of poor outcomes.

#### Machine Learning Algorithm and Features

Our predictive model was constructed using 71 features and the XGBoost classification algorithm [11]. These 71 features are presented in the online multimedia appendix of our previous paper on model building using the UWM data [12]. The features were constructed using the attributes in our UWM data set, which cover diverse aspects such as diagnoses, patient demographics, vital signs, visits, laboratory tests, procedures, and medications. Two exemplary features are the number of days from the patient’s most recent ED visit and the number of asthma diagnoses that the patient received in the previous 12 months. These 71 features were included in every data instance that was inputted to our predictive model.

#### Cutoff Point for Conducting Binary Classification

We set the cutoff point for conducting binary classification at the highest 10% of the risk scores computed by our model. Each patient with a risk score above this cutoff point was projected to have ≥1 asthma hospital visit during the following 12 months.

#### Assessing the Timeliness of the Initial Warnings of Risk Given by Our Model

**Figure 1:**
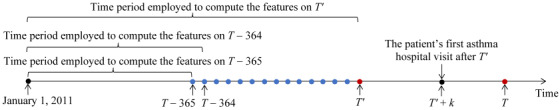
Method of calculating *k*. *T*: the date on which the patient’s first asthma hospital visit in 2019 happened. *T'*: the earliest date between *T* - 365 and *T* - 1 such that by taking the feature values computed on the patient’s historical data up to *T'* as inputs, the model would predict the patient to have ≥1 asthma hospital visit during the 12 months after *T'*. *k*: the number of days of advanced warning that the model gave for the patient for the first time.

Given a predictive model and an asthma patient in 2018 whose first asthma hospital visit in 2019 happened on date *T*, we measured *k*, the number of days in advance that our model gave an initial warning of risk. To compute *k*, we started from *T* - 365 and kept moving forward along the timeline to find the earliest date *T'* (*T* - 365 ≤ *T'* ≤ *T* - 1) such that by taking the feature values computed on the patient’s historical data up to *T'* as inputs, the model would predict the patient to have ≥1 asthma hospital visit during the 12 months after *T'*. In this case, the model warned the patient’s first asthma hospital visit after *T' k* (1 ≤ *k* ≤ *T* - *T'*) days in advance, with *T'* + *k* being the starting date of the patient’s first asthma hospital visit after *T'* (see Figure 1). Otherwise, if the model still predicted no future asthma hospital visit when we reached *T* - 1, the model warned the patient’s asthma hospital visit on *T k* = 0 day in advance. The larger the value of *k*, the more timely the initial warning of risk that the model gave for the patient. *k* reflected how early before a poor outcome occurred the care manager would be prompted for the first time to consider giving the patient preventive interventions. The value of *k* was not affected by any prediction made by the model when the feature values computed based on the patient’s historical data up to a given date after *T'* were taken as inputs.

For our predictive model, we computed *k* for every asthma patient in 2018 who had ≥1 asthma hospital visit at the UWM in 2019. We present the mean and the distribution of *k*.

#### Analyzing False-Positive Predictions Made by Our Model

For each asthma patient in 2018 whom our model erroneously predicted to have ≥1 asthma hospital visit at the UWM in 2019, we used PreManage data to check whether the patient had ≥1 asthma hospital visit outside of the UWM in 2019. We also used the UWM data to check whether the patient had any surrogate indicator of a poor outcome. Surrogate indicators of poor outcomes included a prescription for systemic corticosteroids during the following 12 months (ie, during 2019), any type of visit with a primary or principal diagnosis of asthma exacerbation during the following 12 months (ie, during 2019), and an asthma hospital visit between 13 and 24 months later (ie, during 2020). Systemic corticosteroids are used to treat asthma exacerbation. In addition, if the patient had ≥1 prescription for systemic corticosteroids in 2019, we computed the number of systemic corticosteroids ordered for the patient in 2019 counting multiplicity. This number partially reflected how poorly the patient’s asthma was controlled. We present the distribution of this number.

## Results

### Clinical Characteristics and Demographics of Our Patient Cohort

Multimedia Appendix 1 shows the clinical characteristics and demographics for the UWM asthma patients, presented separately for the period between 2011 and 2017 and for 2018. Every data instance is linked to a distinct index year and patient pair and is used to project the outcome for the patient in the following 12 months. Our previous paper [12] included a detailed comparison of the clinical characteristics and demographics of the 2 sets of patients.

### The Timeliness of Initial Warnings of Risk Given by Our Model

**Figure 2:**
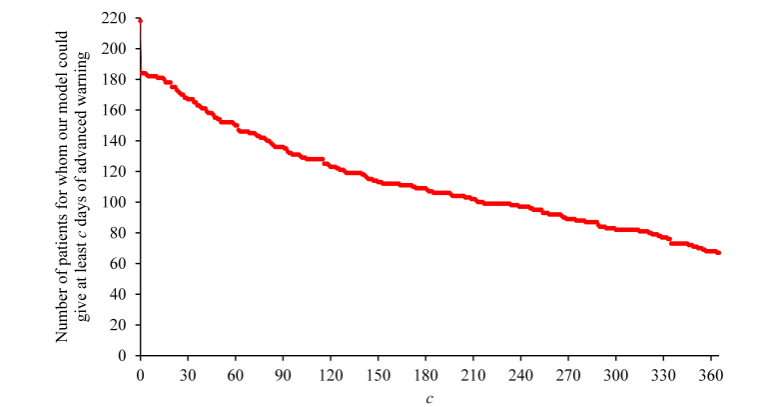
The number of patients for whom our model could give at least *c* days of advanced warning versus *c* (0 ≤ *c* ≤ 365) among the 218 patients with asthma in 2018 who had asthma hospital visits at the University of Washington Medicine in 2019.

Of the 14,644 asthma patients in 2018, 218 (1.49%) had asthma hospital visits at the UWM in 2019. Figure 2 plots the distribution of the number of days in advance that our model gave an initial warning of an asthma hospital visit for every such patient. Our model gave a mean of 190 (SD 150) days of advanced warning. Our model gave an initial warning of risk ≥12 months in advance for 67 of these 218 (30.7%) patients, ≥6 months in advance for 107 of these 218 (49.1%) patients, ≥3 months in advance for 135 of these 218 (61.9%) patients, ≥1 month in advance for 167 of these 218 (76.6%) patients, ≥2 weeks in advance for 181 of these 218 (83%) patients, and ≥1 day in advance for 184 of these 218 (84.4%) patients.

### Breakdown of False-Positive Predictions Made by Our Model

Our model erroneously predicted that 1310 asthma patients in 2018 would have asthma hospital visits at the UWM in 2019 [12]. Table 1 shows the number of these patients who had ≥1 asthma hospital visit outside of the UWM in 2019 or ≥1 surrogate indicator of a poor outcome.

**Figure 3:**
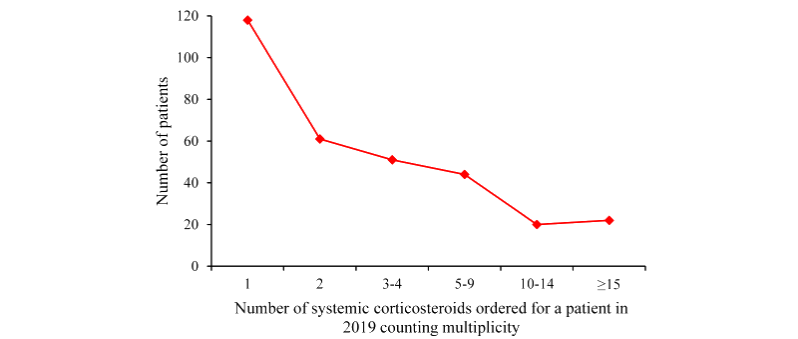
The distribution of the number of systemic corticosteroids ordered for every patient in 2019 counting multiplicity among the 316 asthma patients in 2018 who were erroneously predicted by our model to have ≥1 asthma hospital visit at the University of Washington Medicine in 2019 and also had ≥1 prescription for systemic corticosteroids in 2019.

In total, 316 asthma patients in 2018 were erroneously predicted by our model to have ≥1 asthma hospital visit at the UWM in 2019 and also had ≥1 prescription for systemic corticosteroids in 2019. Figure 3 plots the distribution of the number of systemic corticosteroids ordered for every such patient in 2019 counting multiplicity. The maximum value of this number was 118.

**Table 1:** The number of patients (N=1310) who had ≥1 asthma hospital visit outside of the University of Washington Medicine (UWM) in 2019 or ≥1 surrogate indicator of a poor outcome among the 1310 asthma patients in 2018 whom our model erroneously predicted to have asthma hospital visits at the UWM in 2019.

Outcome	Patients, n (%)
(1) At least 1 prescription for systemic corticosteroids during the following 12 months	316 (24.12)
(2) Any type of visit with a primary or principal diagnosis of asthma exacerbation during the following 12 months	126 (9.62)
(3) Asthma hospital visit between 13 and 24 months later (ie, during 2020)	18 (1.37)
(4) At least 1 asthma hospital visit outside of the UWM during the following 12 months	39 (2.98)
Any of (1), (2), and (3)	358 (27.33)
Any of (1), (2), (3), and (4)	380 (29.01)

## Discussion

### Principal Results

Among the 218 asthma patients in 2018 who had asthma hospital visits at the UWM in 2019, the number of patients for whom our model could give at least *c* days of advanced warning decreased roughly linearly with *c* (0 ≤ *c* ≤ 365) at a fast pace. Our model gave timely risk warnings (eg, ≥3 months in advance) for a large proportion of these 218 asthma patients. Nevertheless, for another large proportion of these 218 asthma patients, our model could not give a timely risk warning. The model either gave a risk warning that was at most a few days in advance or did not predict a patient’s risk even on the day before an asthma hospital visit.

Among the 1310 asthma patients in 2018 whom our model erroneously predicted to have asthma hospital visits at the UWM in 2019, 380 (29.01%) had asthma hospital visits outside of the UWM in 2019 or surrogate indicators of poor outcomes, and hence were reasonable candidates for preventive interventions. Among the 316 of these patients who had ≥1 prescription for systemic corticosteroids in 2019, a large proportion had rather poor asthma control, as reflected by a nontrivial number of systemic corticosteroids that were ordered for these patients in 2019.

### Are the Initial Warnings of Risk Given by Our Model Timely Enough?

A predictive model should identify the risk of having future asthma hospital visits as early as possible in order to give the patient preventive interventions in time to avoid a poor outcome. The time needed for a preventive intervention to take effect varies with the intervention. To the best of our knowledge, there is no consensus on the amount of time needed for a particular preventive intervention or a particular combination of preventive interventions to take effect for averting future asthma hospital visits. Consequently, in this study, we could not compute the exact percentage of patients with future asthma hospital visits for whom our model could give timely risk warnings. Nevertheless, we can shed some light on the rough range of this percentage. In a prior study [27,28], several clinicians gave the opinion that up to 3 months could be needed for any intervention to take effect for averting inpatient stays for an ambulatory care-sensitive, chronic condition such as asthma. For 135 of the 218 (61.9%) asthma patients in 2018 who had asthma hospital visits at the UWM in 2019, our model was able to give an initial warning of risk ≥3 months in advance. Accordingly, we expect that the percentage of patients with future asthma hospital visits for whom our model could give a timely risk warning was at least 61.9%, which is large. On the other hand, for 34 of the 218 (15.6%) asthma patients in 2018 who had asthma hospital visits at the UWM in 2019, our model could not foresee the patient’s risk even on the day before the visit. Thus, the percentage of patients with future asthma hospital visits for whom our model could not give a timely risk warning was at least 15.6%, which is also large. Combining these two findings, we estimate that the percentage of patients with future asthma hospital visits for whom our model could give a timely risk warning was somewhere between 61.9% and 84.4%. Thus, there is still significant room for improving our model to give more timely risk warnings.

### Potential Impact of False-Positive Predictions Made by Our Model

We previously developed an automated method to supply rule-style explanations for the predictions that an arbitrary machine learning model makes on tabular data and to suggest tailored interventions [32,33]. Whenever our model gave a risk warning for a patient, we could use this method to help clinicians decide whether the patient should be enrolled in a care management program, should receive other less-expensive preventive interventions, or did not need any preventive intervention. For 134 of the 153 (87.6%) asthma patients in 2018 whom our model accurately predicted to have asthma hospital visits at the UWM in 2019, our method supplied rule-style explanations for the predictions made by the model [32]. Each such explanation included ≥1 modifiable risk factor and linked to ≥1 intervention [32]; nevertheless, the situation could be different for other prediction targets or health care systems.

We found that among the 1310 asthma patients in 2018 whom our model erroneously predicted to have asthma hospital visits at the UWM in 2019, 380 (29.01%) had asthma hospital visits outside of the UWM in 2019 or surrogate indicators of poor outcomes. These patients could have benefited from the information provided by our automated explanation method. For the other 930 of the 1310 (70.99%) asthma patients in 2018 whom our model erroneously predicted to have asthma hospital visits at the UWM in 2019, our model’s predictions could be truly inaccurate, leaving significant room for improving our model’s accuracy. To know how many of these predictions would mislead clinicians into making incorrect intervention decisions, we would need to perform a user study with clinicians. This is left as an area of interest for future work.

### Related Work

To the best of our knowledge, no prior study has used either surrogate indicators of poor outcomes or future asthma hospital visits at other hospitals to analyze the false-positive predictions made by a predictive model for asthma hospital visits. Also, no prior study has assessed the timeliness of the initial warnings of risk given by such a model. For predicting *Clostridium difficile* infection during an inpatient stay, Wiens et al [34] measured the number of days of advanced warning that a model gave on the patient. For predicting the total amount of donations that a fundraiser could obtain on a medical crowdfunding platform, Wang et al [35] measured the prediction timeliness based on the number of days of input data that a model needed in order to produce predictions within a certain percentage error rate and with a given level of confidence. For predicting the onset of sepsis, Guan et al [36] and Lauritsen et al [37] showed how model accuracy varied by the amount of time from when the model made a prediction to when sepsis occurred. Sepsis is an acute condition, whereas asthma is a chronic condition.

### Limitations

This study has 5 limitations. First, this study was performed in a single health care system. In the future, we plan to use data from other health care systems to perform similar error and timeliness analyses on predicting asthma hospital visits [38,39].

Second, this study shows that many false-positive predictions made by our model could be truly inaccurate. While this study did not examine the factors that could have caused our model to make incorrect predictions, future work to investigate these factors could help improve model performance.

Third, although the PreManage data set covers every hospital in Washington State and many other American hospitals outside of Washington State, the data set does not cover every hospital in the United States. Consequently, our computational results on asthma hospital visits outside of the UWM in 2019 might have missed a small number of asthma patients in 2018 who had asthma hospital visits in 2019 that were outside of the UWM and whose data were unavailable in PreManage.

Fourth, our 3 surrogate indicators of poor outcomes were computed based on the UWM data. Consequently, our computational results for these surrogate indicators missed the asthma patients in 2018 who had surrogate indicators of poor outcomes outside of the UWM.

Fifth, this study computed the number of days in advance that our model gave an initial warning of an asthma hospital visit for a patient. This number reflected how early before a poor outcome a care manager could be prompted for the first time to consider giving the patient preventive interventions. However, it is currently unknown how likely the care manager would take action after receiving such a warning. This is worth studying in future work.

### Conclusions

This study analyzed the errors and timeliness of the risk warnings given by our model for predicting asthma hospital visits. Our results show that our model gave timely risk warnings for most asthma patients with poor outcomes. We found that 380 of the 1310 (29.01%) asthma patients for whom our model gave false-positive predictions had asthma hospital visits outside of our health care system during the following 12 months or surrogate indicators of poor outcomes, and hence were reasonable candidates for preventive interventions. There is thus still significant room for improving our model to give more accurate and more timely risk warnings, such as by using predictive and comprehensible temporal features semiautomatically extracted from longitudinal medical data [35,40,41].

## Appendix

Multimedia Appendix 1 The summary statistics of the clinical characteristics and the demographics of the University of Washington Medicine patients with asthma.

## Abbreviations

ED: emergency department
ICD: International Classification of Diseases
UWM: University of Washington Medicine
XGBoost: extreme gradient boosting
