# Supplementary material for: Error and Timeliness Analysis for Using Machine Learning to Predict Asthma Hospital Visits: Retrospective Cohort Study
Source: JMIR Med Inform. 2022 Jun 8;10(6):e38220. doi: 10.2196/38220 (PMC9218884; doi:10.2196/38220)
Supplement: Multimedia Appendix 1 [file medinform_v10i6e38220_app1.pdf]

Appendix

Table 1. The summary statistics of the clinical characteristics and the demographics of the UWM patients with asthma during 2011-2017.

Variable	Data instances tied to asthma hospital visits at the UWM in the successive 12 months (*N*=1,184), *n* (%)	Data instances tied to no asthma hospital visit at the UWM in the successive 12 months (*N*=67,060), *n* (%)	Data instances (*N*=68,244), *n* (%)
**Age (years)**			
<40	466 (39.36)	22,993 (34.29)	23,459 (34.38)
40 to 65	583 (49.24)	33,306 (49.67)	33,889 (49.66)
65+	135 (11.40)	10,761 (16.05)	10,896 (15.97)
**Gender**			
Male	551 (46.54)	23,647 (35.26)	24,198 (35.46)
Female	633 (53.46)	43,413 (64.74)	44,046 (64.54)
**Race**			
White	507 (42.82)	47,240 (70.44)	47,747 (69.97)
Black or African American	520 (43.92)	7,900 (11.78)	8,420 (12.34)
Asian	96 (8.11)	5,625 (8.39)	5,721 (8.38)
American Indian or Alaska native	32 (2.70)	1,326 (1.98)	1,358 (1.99)
Native Hawaiian or other Pacific islander	14 (1.18)	659 (0.98)	673 (0.99)
Unknown or not reported	15 (1.27)	4,310 (6.43)	4,325 (6.34)
**Ethnicity**			
Non-Hispanic	1,062 (89.70)	55,247 (82.38)	56,309 (82.51)
Hispanic	82 (6.93)	3,444 (5.14)	3,526 (5.17)
Unknown or not reported	40 (3.38)	8,369 (12.48)	8,409 (12.32)
**Insurance**			
Private	424 (35.81)	39,585 (59.03)	40,009 (58.63)
Public	756 (63.85)	28,031 (41.80)	28,787 (42.18)
Self-paid or charity	65 (5.49)	1,301 (1.94)	1,366 (2.00)
**Number of years since the first asthma-related visit in the UWM data set**			
≤3	986 (83.28)	59,887 (89.30)	60,873 (89.20)
>3	198 (16.72)	7,173 (10.70)	7,371 (10.80)
**Asthma medication prescription**			
Short-acting inhaled beta-2 agonist	1,010 (85.30)	46,798 (69.79)	47,808 (70.05)
Inhaled corticosteroid	626 (52.88)	28,263 (42.15)	28,889 (42.33)
Long-acting beta-2 agonist and inhaled corticosteroid combination	499 (42.15)	21,516 (32.08)	22,015 (32.26)
Systemic corticosteroid	614 (51.86)	18,085 (26.97)	18,699 (27.40)
Long-acting beta-2 agonist	374 (31.59)	11,919 (17.77)	12,293 (18.01)
Leukotriene modifier	201 (16.98)	7,970 (11.88)	8,171 (11.97)
Mast cell stabilizer	4 (0.34)	43 (0.06)	47 (0.07)
**Comorbidity**			
Anxiety or depression	372 (31.42)	19,513 (29.10)	19,885 (29.14)
Gastroesophageal reflux	238 (20.10)	12,053 (17.97)	12,291 (18.01)
Allergic rhinitis	172 (14.53)	11,277 (16.82)	11,449 (16.78)
Obesity	177 (14.95)	7,668 (11.43)	7,845 (11.50)
Sinusitis	89 (7.52)	7,172 (10.69)	7,261 (10.64)
Sleep apnea	88 (7.43)	4,468 (6.66)	4,556 (6.68)
Eczema	66 (5.57)	3,825 (5.70)	3,891 (5.70)
Chronic obstructive pulmonary disease	133 (11.23)	3,693 (5.51)	3,826 (5.61)
Cystic fibrosis	1 (0.08)	60 (0.09)	61 (0.09)

Bronchopulmonary dysplasia	0 (0.00)	1 (0.00)	1 (0.00)
**Smoking status**			
Former smoker	221 (18.67)	15,309 (22.83)	15,530 (22.76)
Current smoker	255 (21.54)	13,826 (20.62)	14,081 (20.63)
Never smoker or unknown	708 (59.80)	37,925 (56.55)	38,633 (56.61)

Table 2. The summary statistics of the clinical characteristics and the demographics of the UWM patients with asthma in 2018.

Variable	Data instances tied to asthma hospital visits at the UWM in the successive 12 months (*N*=218), *n* (%)	Data instances tied to no asthma hospital visit at the UWM in the successive 12 months (*N*=14,426), *n* (%)	Data instances (*N*=14,644), *n* (%)
**Age (years)**			
<40	77 (35.3)	4,746 (32.90)	4,823 (32.94)
40 to 65	111 (50.9)	6,683 (46.33)	6,794 (46.39)
65+	30 (13.8)	2,997 (20.78)	3,027 (20.67)
**Gender**			
Male	100 (45.9)	5,138 (35.62)	5,238 (35.77)
Female	118 (54.1)	9,288 (64.38)	9,406 (64.23)
**Race**			
White	110 (50.5)	10,103 (70.03)	10,213 (69.74)
Black or African American	79 (36.2)	1,491 (10.34)	1,570 (10.72)
Asian	18 (8.3)	1,307 (9.06)	1,325 (9.05)
American Indian or Alaska native	8 (3.7)	273 (1.89)	281 (1.92)
Native Hawaiian or other Pacific islander	2 (0.9)	129 (0.89)	131 (0.89)
Unknown or not reported	1 (0.5)	1,123 (7.78)	1,124 (7.68)
**Ethnicity**			
Non-Hispanic	196 (89.9)	12,370 (85.75)	12,566 (85.81)
Hispanic	20 (9.2)	830 (5.75)	850 (5.80)
Unknown or not reported	2 (0.9)	1,226 (8.50)	1,228 (8.39)
**Insurance**			
Private	108 (49.5)	10,692 (74.12)	10,800 (73.75)
Public	182 (83.5)	7,841 (54.35)	8,023 (54.79)
Self-paid or charity	25 (11.5)	459 (3.18)	484 (3.31)
**Number of years since the first asthma-related visit in the UWM data set**			
≤3	124 (56.9)	10,442 (72.38)	10,566 (72.15)
>3	94 (43.1)	3,984 (27.62)	4,078 (27.85)
**Asthma medication prescription**			
Short-acting inhaled beta-2 agonist	164 (75.2)	9,540 (66.13)	9,704 (66.27)
Inhaled corticosteroid	108 (49.5)	6,069 (42.07)	6,177 (42.18)
Long-acting beta-2 agonist and inhaled corticosteroid combination	83 (38.1)	4,425 (30.67)	4,508 (30.78)
Systemic corticosteroid	120 (55.1)	4,043 (28.03)	4,163 (28.43)
Long-acting beta-2 agonist	62 (28.4)	2,456 (17.02)	2,518 (17.19)
Leukotriene modifier	46 (21.1)	2,130 (14.77)	2,176 (14.86)
Mast cell stabilizer	1 (0.5)	13 (0.09)	14 (0.10)
**Comorbidity**			
Anxiety or depression	62 (28.4)	4,284 (29.70)	4,346 (29.68)
Gastroesophageal reflux	46 (21.1)	2,611 (18.10)	2,657 (18.14)
Allergic rhinitis	26 (11.9)	2,069 (14.34)	2,095 (14.31)
Obesity	25 (11.5)	1,579 (10.95)	1,604 (10.95)
Sinusitis	15 (6.9)	1,357 (9.41)	1,372 (9.37)

Sleep apnea	24 (11.0)	1,475 (10.22)	1,499 (10.24)
Eczema	11 (5.1)	732 (5.07)	743 (5.07)
Chronic obstructive pulmonary disease	30 (13.8)	902 (6.25)	932 (6.36)
Cystic fibrosis	0 (0.0)	17 (0.12)	17 (0.12)
Bronchopulmonary dysplasia	0 (0.0)	4 (0.03)	4 (0.03)
**Smoking status**			
Former smoker	41 (18.8)	3,453 (23.94)	3,494 (23.86)
Current smoker	49 (22.5)	3,193 (22.13)	3,242 (22.14)
Never smoker or unknown	128 (58.7)	7,780 (53.93)	7,908 (54.00)
